# Karyotypes of Manatees: New Insights into Hybrid Formation (*Trichechus inunguis* × *Trichechus m. manatus*) in the Amazon Estuary

**DOI:** 10.3390/genes13071263

**Published:** 2022-07-16

**Authors:** Renata C. R. Noronha, Bruno R. R. Almeida, Monique C. S. Chagas, Flávia S. Tavares, Adauto L. Cardoso, Carlos E. M. C. Bastos, Natalia K. N. Silva, Alex G. C. M. Klautau, Fábia O. Luna, Fernanda L. N. Attademo, Danielle S. Lima, Luiz A. Sabioni, Maria I. C. Sampaio, Jairo Moura Oliveira, Luís Adriano Santos do Nascimento, Cesar Martins, Marcelo R. Vicari, Cleusa Y. Nagamachi, Julio C. Pieczarka

**Affiliations:** 1 Laboratório de Citogenética, Centro de Estudos Avançados da Biodiversidade, Instituto de Ciências Biológicas, Universidade Federal do Pará, Belém 66075-110, PA, Brazil; brunorafa18@gmail.com (B.R.R.A.); moniquecschagas@gmail.com (M.C.S.C.); flaviatavares.ft.77@gmail.com (F.S.T.); carlosedu.bastos@gmail.com (C.E.M.C.B.); cleusanagamachi@gmail.com (C.Y.N.); juliopieczarka@gmail.com (J.C.P.); 2 Campus Itaituba, Instituto Federal de Educação, Ciência e Tecnologia do Pará, Itaituba 68183-300, PA, Brazil; 3 Laboratório Genômica Integrativa, Departamento de Biologia Estrutural e Funcional, Instituto de Biociências de Botucatu, Universidade Estadual Paulista, Botucatu 18618-970, SP, Brazil; adautolimacardoso@gmail.com (A.L.C.); cesar.martins@unesp.br (C.M.); 4 Campus Tucuruí, Universidade do Estado do Pará, Tucuruí 68455-210, PA, Brazil; natalianascimento1108@gmail.com; 5 Centro Nacional de Pesquisa e Conservação da Biodiversidade Marinha do Norte, Instituto Chico Mendes de Conservação da Biodiversidade, Belém 66635-110, PA, Brazil; alexgk@globo.com; 6 Centro Nacional de Pesquisa e Conservação de Mamíferos Aquáticos, Instituto Chico Mendes de Conservação de Biodiversidade, Santos 11050-031, SP, Brazil; fabia.luna@icmbio.gov.br (F.O.L.); niemeyerattademo@yahoo.com.br (F.L.N.A.); 7 Departamento de Zoologia, Programa de Pós-Graduação em Biologia Animal/PPBA, Laboratório de Ecologia Comportamento e Conservação/LECC, Universidade Federal de Pernambuco/UFPE, Recife 50670-901, PE, Brazil; 8 Grupo de Pesquisa em Mamíferos Aquáticos Amazônicos, Instituto de Desenvolvimento Sustentável Mamirauá, Estrada do Bexiga, Tefé 69553-225, AM, Brazil; limadanielle@terra.com.br (D.S.L.); luizsabioni@gmail.com (L.A.S.); 9 Rede de Pesquisa e Conservação de Sirênios no Estuário Amazônico, Macapá 68903-197, AP, Brazil; 10 Campus Porto Grande, Instituto Federal de Educação Ciência e Tecnologia do Amapá, Rodovia BR 210, Km 103, s/n, Zona Rural, Porto Grande 68997-000, AP, Brazil; 11 Instituto de Estudos Costeiros, Campus Bragança, Universidade Federal do Pará, Bragança 68600-000, PA, Brazil; iracilda.sampaio@gmail.com; 12 Zoological Park of Santarém, ZOOUNAMA, Universidade da Amazônia, Santarém 68030-150, PA, Brazil; jairomoura@hotmail.com; 13 Laboratório de Óleos da Amazônia, Universidade Federal do Pará, Belém 66075-110, PA, Brazil; adrlui1@yahoo.com.br; 14 Departamento de Biologia Estrutural, Molecular e Genética, Universidade Estadual de Ponta Grossa, Ponta Grossa 84030-900, PR, Brazil; vicarimr@yahoo.com.br

**Keywords:** karyotype, hybridization, repetitive DNA, sirenia

## Abstract

Great efforts have been made to preserve manatees. Recently, a hybrid zone was described between *Trichechus inunguis* (TIN) and the *Trichechus manatus manatus* (TMM) in the Amazon estuary. Cytogenetic data on these sirenians are limited, despite being fundamental to understanding the hybridization/introgression dynamics and genomic organization in *Trichechus*. We analyzed the karyotype of TMM, TIN, and two hybrid specimens (“Poque” and “Vitor”) by classical and molecular cytogenetics. G-band analysis revealed that TMM (2n = 48) and TIN (2n = 56) diverge by at least six Robertsonian translocations and a pericentric inversion. Hybrids had 2n = 50, however, with Autosomal Fundamental Number (FNA) = 88 in “Poque” and FNA = 74 in “Vitor”, and chromosomal distinct pairs in heterozygous; additionally, “Vitor” exhibited heteromorphisms and chromosomes whose pairs could not be determined. The U2 snDNA and Histone H3 multi genes are distributed in small clusters along TIN and TMM chromosomes and have transposable Keno and Helitron elements (TEs) in their sequences. The different karyotypes observed among manatee hybrids may indicate that they represent different generations formed by crossing between fertile hybrids and TIN. On the other hand, it is also possible that all hybrids recorded represent F1 and the observed karyotype differences must result from mechanisms of elimination.

## 1. Introduction

The Trichechidae family is a monotypic taxon currently represented by the genus *Trichechus*, which comprises three species of manatees: *T. inunguis* (Amazonian manatee), freshwater, with distribution in the Amazon Basin; *Trichechus senegalensis* (African manatee), marine, widely found off the coast of Senegal and Angola; and *T. manatus*, marine, distributed from Florida to the coast of northeastern Brazil [1]. Cranial morphometric studies revealed the existence of two subspecies for *T. manatus*: *T. manatus latirostris* (Florida manatee) and *T. manatus manatus* (Antillean manatee, distributed in the Caribbean, Central America, and Brazil) [2]. To date, only *T. manatus latirostris* (2n = 48), *T. manatus manatus* (2n = 48), and *T. inunguis* (2n = 56) have briefly described karyotypes, with pair 20 carrying the nucleolus organizer region (NOR) in the terminal region and exclusively pericentromeric constitutive heterochromatin [3,4,5]. Despite the existence of G-banded karyotypes and total chromosome probes for genomic mapping of the *Trichechus* species, no comparative analysis has been performed to infer evolutionary relationships within the genus [6].

Genetic analysis confirmed the existence of a hybrid zone between *T.m manatus* and *T. inunguis*, which extends from the coast of Guyana to the mouth of the Amazon River, where these two species cohabit due to feeding needs and favorable abiotic factors [7,8]. The first probable manatee hybrid to be studied cytogenetically, “Poque”, corresponds to a male rescued in clandestine captivity in the state of Amapá (Brazil), for which Vianna et al. [8] described 2n = 50, and proposed to be the result of crossing a hybrid female and a *T. manatus* male, however, they did not provide detailed karyotyping or analyses of specific chromosomal regions about this individual. Subsequently, Oliveira et al. [9] described two other hybrid individuals, 2n = 49, both carriers of certain pairs of the karyotype in a heterozygous condition for fusion/fission. Mitochondrial DNA and microsatellite data were not fully effective in detecting hybrids among the studied sirenians [7,8,10]. In this context, chromosomal analysis can be an excellent tool for identifying and evaluating the fertility/viability of individuals involved in the hybridization process and existing generations from F1.

Repetitive DNA sequences comprise a considerable portion of the eukaryotes genome and have been implicated in the divergence processes of hybrids and their progenitors [11]. They are mainly represented by multigene families (rDNAs, histone genes, U snDNA, etc.), satellite DNAs, microsatellites, and transposable elements [12]. In the *Trichechus* genome, the centromeric satellite DNA TMAsat [12,13], microsatellites [9], and the mobile element Long Interspersed Nuclear Element (LINE-1) [14] were previously identified (and mapped in chromosomes). The genomic shock caused by the union of genomes in interspecific hybrids can promote changes in gene expression, the occurrence of chromosomal rearrangements, and epigenetic alterations [15]. The genome of interspecific hybrids normally shows high expression and proliferation of transposable elements, which can interrupt genes, or be inserted into regulatory regions and modulate their expression [11,15]. The association of multigene families with transposable elements and their involvement in DNA recombination and repair events may favor their spread to distinct chromosomal loci in both parental and hybrid genomes [16]. The U2 snDNA, for example, presents itself in a pair of homologs in most organisms, however, in some fish, its chromosomal distribution was recorded in a dispersed way in several elements of the karyotype [17].

In the present study, we describe the karyotype of two *Trichechus* hybrids, and compare their banding patterns and repetitive DNA distribution with *T. m. manatus* and *T. inunguis*, to clarify the rearrangements involved in the karyotypic differentiation of these species, their genomic organization, and evolutionary relationships.

## 2. Materials and Methods

### 2.1. Samples

The samples used in the present study consisted of three male and three female specimens of *T. inunguis* (TIN), from the captivity program of the Faculdades Integradas do Tapajós, in Santarém, Pará State, Brazil, and two males and two females *T. m. manatus* (TMM), from the National Center for Research and Conservation of Aquatic Mammals (CMA), in Itamaracá, State of Pernambuco, Brazil. Two probable hybrids were used in the present study: a male named “Poque”, who lives in captivity at the CMA, and another male specimen known as “Vitor”, rescued by the Instituto do Meio Ambiente e Recursos Renováveis, both from the State of Amapá, Brazil. SISBIO collection authorization (Number: 44915-1). All applicable international, national, and/or institutional guidelines for the care and use of animals were followed. The Cytogenetics Laboratory of the Universidade Federal do Pará has a license nº 19/2003 from the Ministry of the Environment for the transport of samples, and a license 52/2003 for the use of samples for research. The animal study protocol was approved by the Research Ethics Committee of the Universidade Federal do Pará (Permission 68/2015).

### 2.2. Chromosomal Preparations and Karyotype Analysis

Chromosomal preparations were obtained employing temporary lymphocyte culture, according to the protocol developed by Moorhead et al. [18], with the following modifications: 0.5 mL of whole blood were seeded in a 5 mL RPMI-1640 medium for 96 h. One hour before the incubation process, Karyomax colcemid 10μg/mL was added. The samples were submitted to hypotonic treatment with KCL 0.075 M for 20 min and fixed in Carnoy solution (1 acetic acid: 3 methanol). The best metaphases were photographed and used for classical cytogenetic analysis. The detection of the constitutive heterochromatin distribution pattern (C-banding) was obtained by the protocol described by Summer [19]. The G-banding pattern was performed using Seabright’s protocols [20]. The best metaphases were selected for karyotype assembly according to Levan et al. [21], in Adobe Photoshop software version CS6.

### 2.3. DNA Extraction and Isolation of Repetitive Sequences

TMM genomic DNA was extracted from blood samples according to Sambrook et al. [22] and used for isolation of repetitive DNAs by Polymerase Chain Reaction (PCR). Vertebrate telomeric repeats were amplified with complementary primers (TTAGGG)n and (CCCTAA)n, in the absence of template DNA according to Ijdo et al. [23]. Isolation of other repetitive sequences was performed using the following sets of primers: F 5′-ATGGCTCGTACCAAGCAGAC(ACG)GC-3′ and R5′-ATATCCTT(AG)GGCAT(AG) AT(AG)GTGAC-3 ′ for Histone H3 [24]; F5′-TCTCGGCCT(AT)(AT)TGGCTAA-3′ and R5′-G(AC)GGTA(GC)TGCAATACCGG-3′, for U2 snDNA [25]; 5′-ATTCTRTTCCATTGGTCTA-3′ and 5′-CCATGCTCATSGATTGG-3′, for LINE-1 transposable element [14]. 45S rDNA probes were produced using plasmid pTa71, which contains the complete sequence of this rDNA isolated from the genome of *Triticum aestivum* [26].

The Polymerase Chain Reaction consisted of 16.05 μL of sterile water, 2.5 μL buffer 10 × 0.7 μL de MgCl_2_ (50 mM), 1.5 μL of dNTP (8 mM), 100 ng of total DNA, 1.5 µL of each gene-specific primer and 0.25 µL of 1U Taq Polymerase. The amplification cycles and the temperatures used were: 1 cycle of 95 °C for 5 min; 30 cycles at 95 °C for 40 s, 52–58 °C for 40 s (variable by primer), and 2 min at 72 °C; a cycle of 72 °C for 10 min; hold at 4 °C.

### 2.4. Sequence Analysis

PCR products were purified using a GenElute PCR Clean-Up Kit (Sigma-Aldrich, Burlington, VT, USA) and sequenced using an ABI 3500 genetic analyzer (Applied Biosystems, Foster City, CA, USA). The sequences were edited and analyzed using Geneious 7.1.3 software [27], and their identities were confirmed using the CENSOR tool for repeated sequences (Girinst) [28] and BLASTn (NCBI).

### 2.5. Fluorescent In Situ Hybridization (FISH)

The preparations obtained from the PCRs were labeled by nick translation with biotin (BioNick Labeling System kit, Invitrogen, Waltham, MA, USA), or with digoxigenin (DIG-Nick Translation Mix kit, Roche), according to the manufacturers’ instructions. Fluorescent in situ hybridization was performed following the protocol described by Yang et al. [29]. Signals were detected with Avidin-Cy3 or Anti-digoxigenin-FITC. Chromosomes were stained with DAPI (4,6-diamidino-2-phenylindole) containing antifading Vectashield (Vector, Burlingame, CA, USA). FISH images were captured using an AxioCam MRm CCD camera (Nikon, Tokyo, Japan) attached to a Nikon H550S Epifluorescence microscope (Nis-Elements AR program (Nikon, Tokyo, Japan)).

## 3. Results

### 3.1. Karyotype Description and Comparisons

**Figure 1:**
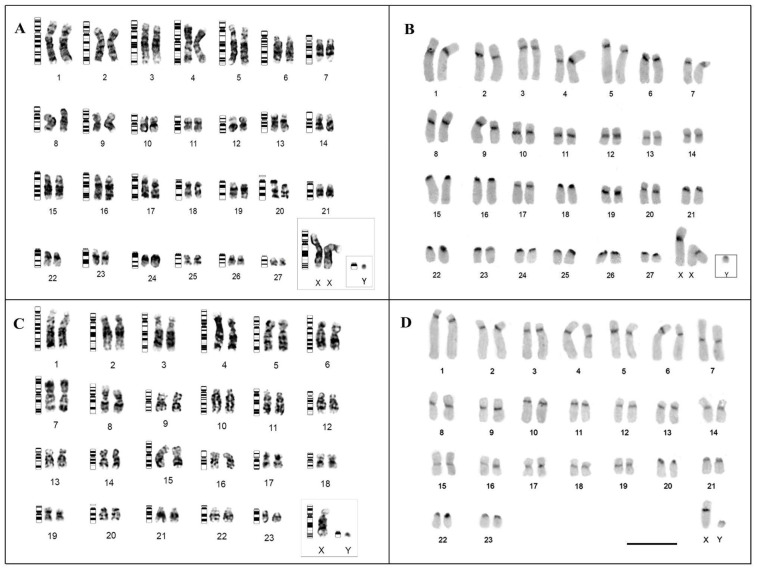
Karyotype of TIN (**A**,**B**) and TMM (**C**,**D**), after G-banding (**A**,**C**) and C-banding (**B**,**D**). In (**A**,**C**), an ideogram shows the pattern of G bands next to each chromosome pair. Bar 10 µm.

The analyzed TIN specimens presented a karyotype with 2n = 56 and an autosomal fundamental number (NFa) equal to 92, composed of 19 pairs of metacentric/submetacentric chromosomes (pairs 1–14,17–18,20–21,23), and eight acrocentric pairs (pairs 15–16,19,22,24–27) (Figure 1A); the sex-determination system is of the XY type, with submetacentric X and acrocentric Y (Figure 1A). In turn, TMM specimens presented 2n = 48 and Nfa = 84, with 19 pairs of metacentric/submetacentric morphology (pairs 1 to 19), and four pairs of acrocentric chromosomes (pairs 20 to 23) (Figure 1C); the sex pair is of the XY type, with submetacentric X and acrocentric Y (Figure 1C). Through the G-banding analysis, it was possible to identify the pairing of all chromosomes (Figure 1A,C). Constitutive heterochromatin (HC) was identified in the pericentromeric region of all pairs in both species (Figure 1B,D).

**Figure 2:**
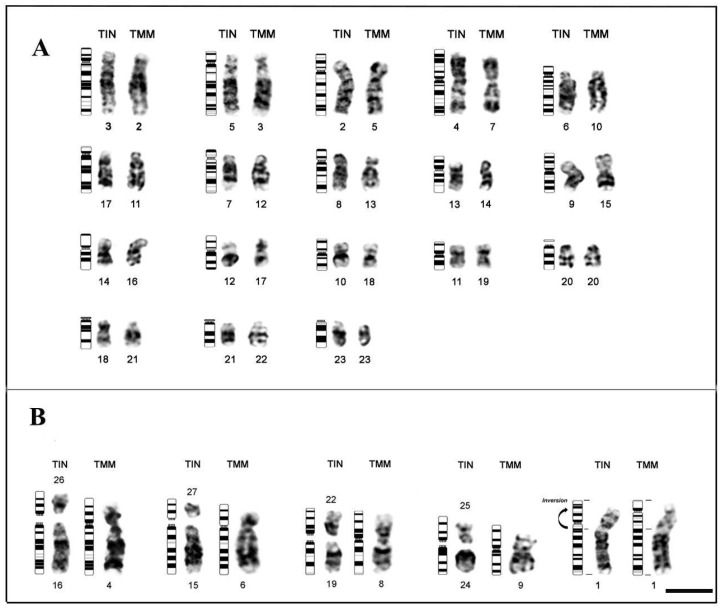
Comparative analysis by G-banding between TIN and TMM: (**A**) possible chromosomal homologies; and (**B**) possible chromosomal rearrangements. Bar 10 µm.

Analysis between TIN and TMM, using G-banding patterns, allowed the identification of possible chromosomal homologies between these two sirenians species (Figure 2); thus, when making comparisons between the karyotypes of both species, we observed 18 pairs of conserved chromosomes: pair 3 of TIN (3TIN) shows homeology with chromosome 2 of TMM (2TMM) (3TIN-2TMM); 5TIN-3TMM; 2TIN-5TMM; 4TIN-7TMM; 6TIN-10TMM; 17TIN-11TMM; 7TIN-12TMM; 8TIN-13TMM; 13TIN-14TMM; 9TIN-15TMM; 14TIN-16TMM; 12TIN-17TMM; 10TIN-18TMM; 11TIN-19TMM; A20TIN-20TMM; 18TIN-21TMM; 21TIN-22TMM; 23TIN-23TMM (Figure 2A). Likewise, through the G-banding pattern, some possible structural rearrangements were identified: fusion between pairs 16 and 26 of TIN that correspond to chromosome 4 of TMM; fusion between pairs 15 and 27 of TIN, corresponding to 6 of TMM; fusion between pairs 19 and 22 of TIN, corresponding to 8 of TMM; and the merger between pairs 24 and 25 of TIN, corresponding to 9 of TMM; and a pericentric inversion on the short arm of TIN chromosome 1, corresponding to TMM chromosome 1 (Figure 2B).

**Figure 3:**
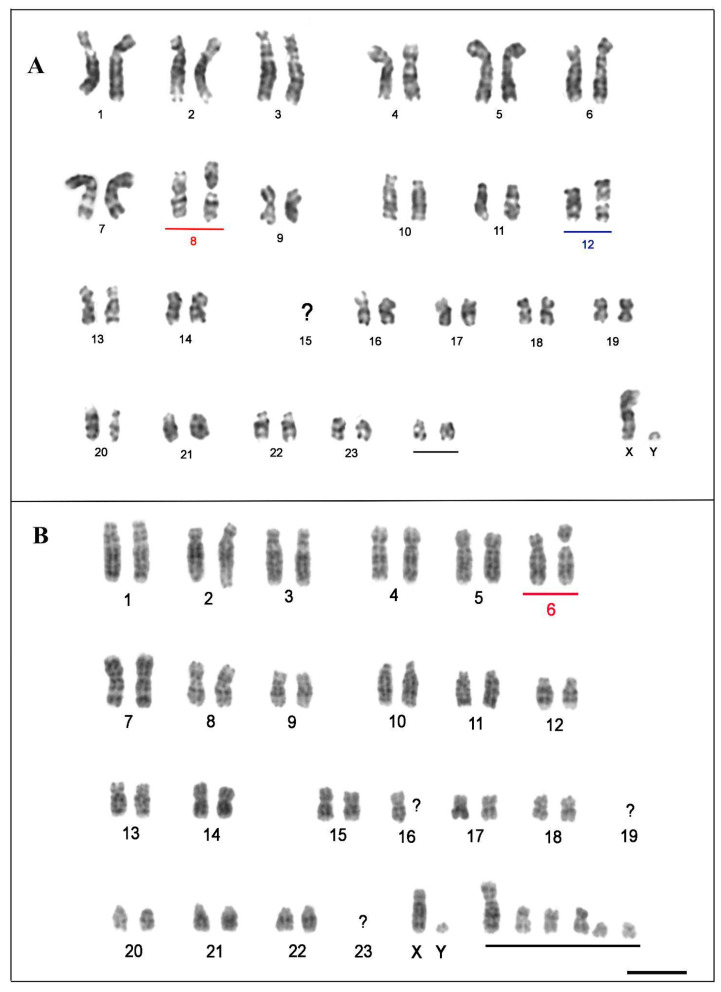
G-banded karyotype of hybrid specimens of manatee: (**A**). “Poque”; (**B**). “Vitor”. The blue and red bars indicate probable homologies. The black bars show chromosomes whose pairs could not be determined by the banding pattern. Bar 10 µm.

The karyotypes of probable hybrids were organized in accordance with TMM karyotype. The individuals “Poque” and “Vitor” presented a distinct karyotype from the TIN and TMM species, with 2n = 50 (Figure 3). “Poque” showed NFa = 88, composed of 20 metacentric/submetacentric and 4 acrocentric chromosome pairs, with submetacentric X and acrocentric Y (Figure 3A). Based on the comparative analysis between the G-banding patterns of “Poque”, we identified that the chromosomes of pairs 8 and 12 present fission of one of the homologs, which correspond to two distinct acrocentric chromosomes (Figure 3A). Additionally, chromosomes equivalent to pair 15 of TMM were absent in this specimen (Figure 3A); on the other hand, we detected the presence of an extra pair formed by two acrocentric chromosomes (Figure 3A).

Although “Vitor” also showed 2n = 50, he showed NFa = 74 (Figure 3B). The karyotype of this specimen consists of 22 acrocentrics and 26 metacentric/submetacentric chromosomes (Figure 3B). After G-banding analysis, it was observed that: (1) pair 2 presents size heteromorphism; (2) pair 6 has one of the fissioned homologs, corresponding to two unequally sized acrocentric chromosomes; (3) pairs 19 and 23 are absent; (4) only one of the pair 16 homologs could be determined (Figure 3B). Furthermore, 6 chromosomes did not show homology in terms of the banding pattern with any other element of the karyotype (Figure 3B). The sex chromosome system was of the XY type, with submetacentric X and acrocentric Y (Figure 3B).

### 3.2. Mapping of Repetitive DNA Sequences

**Figure 4:**
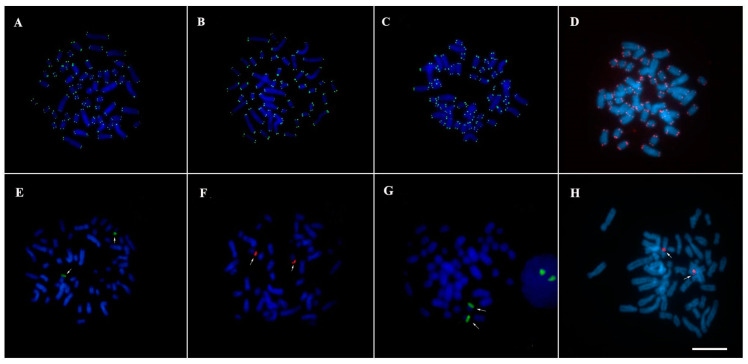
Fluorescent in situ hybridization with probes of telomeric sequences (**A**–**D**) and 45S rDNA (**E**–**H**): (**A**,**E**) *T. inunguis*; (**B**,**F**) *T. m. manatus*; (**C**,**G**) “Poque”; and (**D**,**H**) “Vitor”. Bar 10 µm.

Hybridization signals with telomeric sequence probes (TTAGGG) were observed in the distal regions of all chromosomes in TMM (Figure 4A), TIN (Figure 4B), “Poque” (Figure 4C), and “Vitor” (Figure 4D), without evidence of markings in the interstitial regions (ITS). 45S rDNA sites were recorded in the proximal region of the short arm of the pair 20, in agreement with the nucleolus organizer region (NOR), in TMM (Figure 4E), TIN (Figure 4F), “Poque” (Figure 4G), and “Vitor” (Figure 4H).

**Figure 5:**
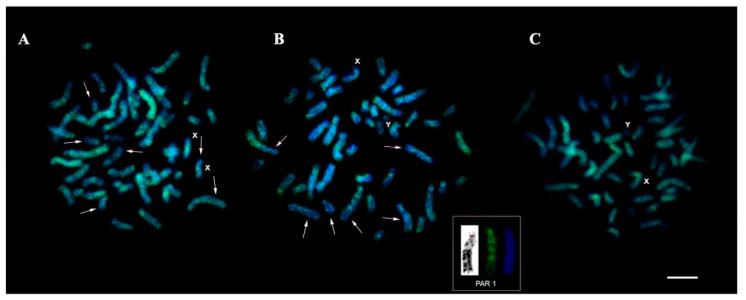
Chromosomal mapping of the transposable element LINE-1: (**A**) *T. inunguis*; (**B**) *T. m. manatus*; the insert indicates the pair 1 chromosome with a LINE-1 pattern similar to bands and compared to the pattern of G bands; and (**C**) “Poque”. Arrows indicate chromosomal regions in TIN and TMM karyotypes with low LINE-1 concentration. Bar 10 µm.

The LINE-1 retrotransposon showed distribution along the length of the chromosomes, with some accumulation in pericentromeric regions in the TIN (Figure 5A), TMM (Figure 5B), and “Poque” (Figure 5C) karyotypes. However, concerning TMM and TIN, “Poque” presented a greater number of LINE-1 signals, completely covering the chromosomes, while in TMM and TIN regions with a low concentration of this transposable element were recorded (Figure 5). In one of the TMM pair 1 homologs (Figure 5B, box), a band-like pattern of LINE-1 organization was recorded (Figure 5B).

**Figure 6:**
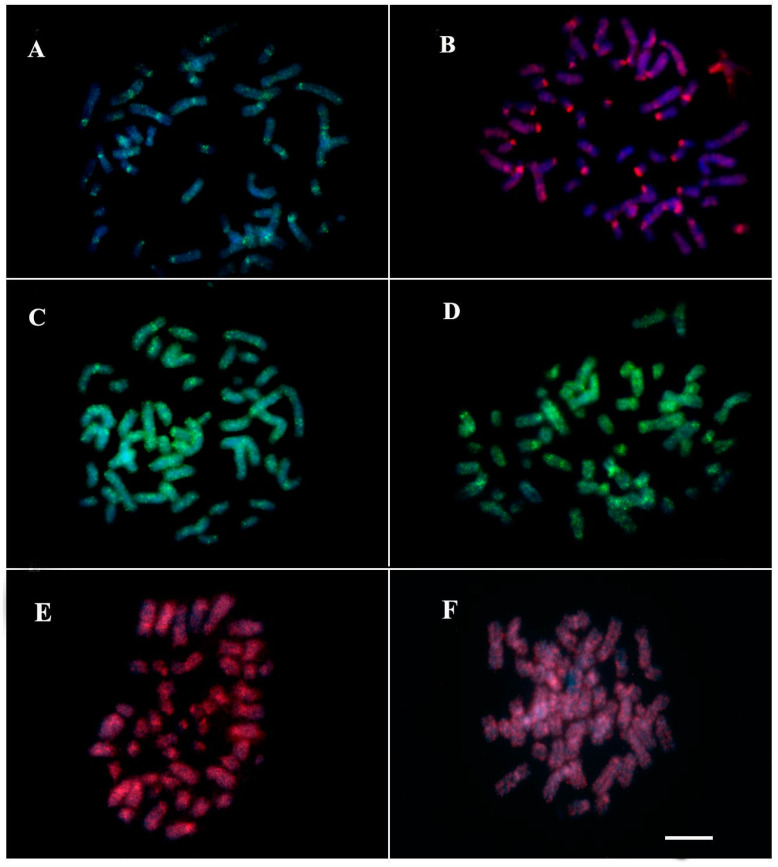
FISH with probe of U2-snDNA (**A**,**B**) and H3 Histone (**C**–**F**) genes; (**A**–**C**) *T. m. manatus*; (**B**–**D**) *T. inunguis*; (**E**) “Poque”; and (**F**) “Vitor”. Bar 10 µm.

**Figure 7:**
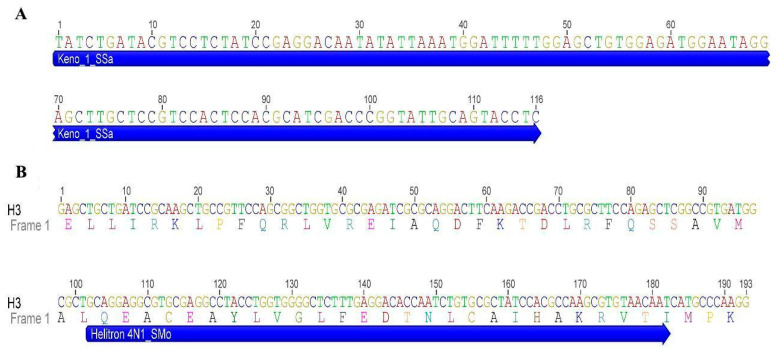
Repetitive DNA sequences isolated from the *Trichechus* genome: (**A**) partial sequence of U2 snRNA with similarity to non-LTR Keno_1_SSa retrotransposon (blue); and (**B**) partial H3 sequence of Trichechus showing a segment similar (102–182 bp) to the Helitron 4N1_SMo transposon (blue).

The results of chromosome mapping with U2 snDNAprobes (Figure 6A,B) and Histone H3 (Figure 6C,D) were performed in TMM, TIN and hybrids. Both sirenians showed dispersed signals along all chromosomes, with a higher concentration of this multigene in pericentromeric regions (Figure 6). Sequence analyses demonstrate that U2 snRNA shows 98% of identity and e-value 8e-48 with *Cavia porcellus* U2 spliceosomal RNA (LOC111758179), ncRNA. Using the Censor tool, the sequence has 86,21% similarity with retrotransposon nonLTR/Tx-1, Keno-1SSa of Atlantic salmon (Figure 7A). The H3 sequence demonstrated 97% identity and e-value 3e-84 with *Camelus ferus* histone H3.1 (LOC102506474), mRNA. In the Censor tool, the segment ranging from 102–182 was 69.51% similar to the DNA transposon Helitron 4N1_SMo of *Selaginella moellendorffii* (Figure 7B).

## 4. Discussion

The cytogenetic analysis of the present study showed karyotypes with diploid numbers previously described for both TIN and TMM species [3,5,30]. However, comparing our fundamental number data (NFa = 92 for TIN and NFa = 84 for TMM) with those described in the literature, we observed differences between populations of the same species. For TIN, for example, Loughman et al. [31] identified NF = 74 for a specimen from Colombia, while Assis et al. [3] described NF = 82 for specimens collected in Manaus (Amazonas, Brazil) and Letícia (Colombia). Fundamental number differences between these populations may be mainly associated with pericentric inversions and demonstrate the existence of geographic variations in the *Trichechus* karyotype. According to phylogenetic analysis by Domning [32] and Vianna et al. [8] TIN is phylogenetically basal in relation to the other species of the genus. Thus, we propose that centric fusions between TIN and TMM may have been the main chromosomal alterations responsible for the reduction of the diploid number 56 to 48. In the present study, although we identified rearrangements by classical cytogenetics, we did not observe interstitial telomeric sites (ITS) in TIN and TMM. The absence of ITS in *Trichechus* may be related to the loss of repeats during the telomere fusion process or the small size of these sequences, to the point of not being detected by the FISH technique [33,34].

A detailed karyotype description of the “Poque” karyotype was performed for the first time in this study using classical and molecular cytogenetic methods. Our analysis confirms the 2n = 50 cited by Vianna et al. [8]. A second specimen collected in the state of Amapá, “Vitor”, also presented 2n = 50. “Poque” and “Vitor” exhibited heterozygous conditions in relation to the morphology of some homologous chromosomes (pairs 8 and 12 in “Poque”; pair 6 in “Vitor”; Figure 3A,B). The two-armed form of these chromosomes is found in TMM, while the acrocentric forms are present in the TIN karyotype. This pattern agrees with the hybrid origin of “Vitor” and “Poque”. Interestingly, pairs 8 and 6 were also recorded as heterozygotes in hybrid individuals with 2n = 49, from the same geographic region [9]. These results suggest a dynamic system of trivalent formation with alternating segregation between elements of pairs 8, 6, and 12 during gametogenesis of hybrid *Trichechus* specimens, and denote a certain fertility rate for these specimens, as suggested by Oliveira et al. [9]. Additionally, our findings suggest “Poque” and “Vitor” as individuals belonging to the F2 generation, resulting from the cross between a hybrid female and a TMM male, as suggested by Vianna et al. [8].

The “Vitor” karyotype showed other cytogenetic differences in relation to “Poque” and the 2n = 49 hybrids [9], such as NFa = 74, heteromorphism of pair 2, the absence of a homolog in pair 16, in addition to the presence of six chromosomes whose pairs could not be determined by the morphology and pattern of G bands, and which may correspond to univalents. We propose that these chromosomal alterations result from the formation of multivalent associations during meiosis I of specimens of the F1 generation (females). Interspecific hybrids resulting from the crossing of highly divergent species in relation to chromosomal structure, commonly present high structural heterozygosity, and formation of chains and meiotic rings [35,36]. Probably, F1 hybrids originated by crossing the two *Trichechus* species analyzed in this article fit this context, since our G-banding comparison between TIN and TMM showed that these sirenians are divergent by at least five Robertsonian rearrangement events and by the pericentric inversion of pair 1. In this case, segregation errors can occur between the pairs involved in the multi-chromosomal association of the F1 hybrid, since they are susceptible to a non-alternating distribution of their components [37]. Other factors that can increase the probability of errors during Anaphase I are variations in the number of copies of repetitive sequences present in the centromere, which can cause segregation problems in gametogenesis in female hybrids, by altering the meiotic drive [38]. Thus, part of the elements of Vitor’s karyotype may correspond to univalents, which originated through the uneven and random distribution of chromosomes during anaphase I in the progenitor hybrid female (F1); univalents may show anaphase delay, and for this reason, tend to be eliminated more frequently [37]; in this case, unbalanced gametes would be formed in large proportion, and the probability of reconstituting the 2n = 50 karyotype would be extremely low in the F3 generation after backcrossing. Thus, “Vitor” may have a greater reduction in fertility compared to “Poque”.

**Figure 8:**
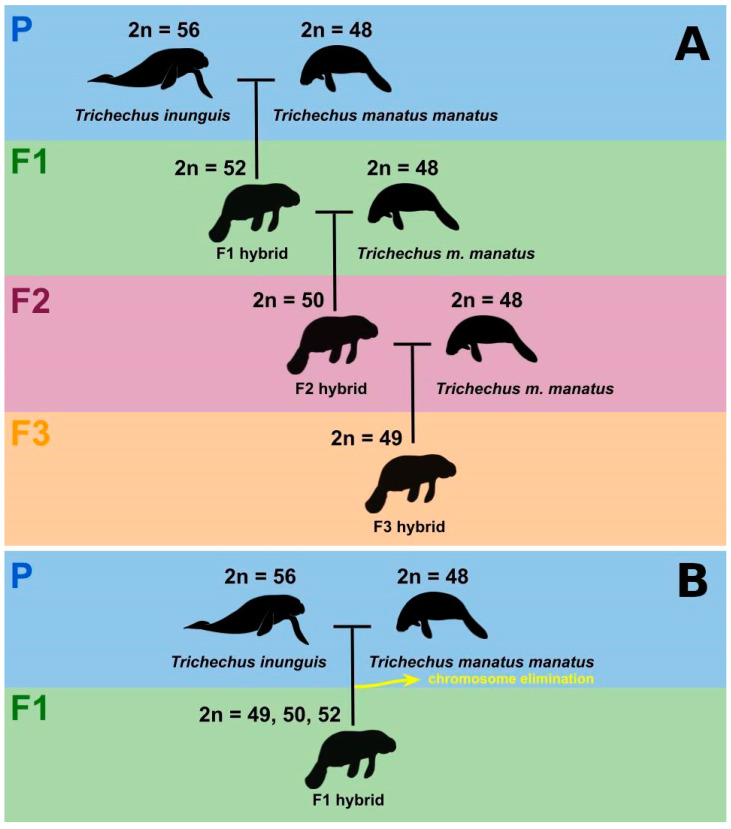
Hypothesis of hybrid formation in *Trichechus* in the Amazon estuary: (**A**) crosses between *T. inunguis* (2n = 56) and *T. m. manatus* (2n = 48) generate the F1 hybrid (2n = 52). Backcross between F1 hybrid and *T. m. manatus* generate F2 hybrid (2n = 50). In turn, interbreeding between F2 hybrids and *T. m. manatus* produces F3 hybrids (2n = 49); and (**B**) crosses between *T. inunguis* (2n = 56) and *T. m. manatus* (2n = 48) generate the F1 hybrids with distinct chromosome numbers (2n = 49, 50, 52) as consequence of chromosome elimination.

The karyotype constitution of the hybrids observed in the present work and those described by De Oliveira et al. [9] indicate the occurrence of several types of chromosome combinations in gametes of hybrids. Moreover, our comparisons reveal that the hybrids described by these authors must be more related with “Poque” than “Vitor”. Considering that F1 hybrids between TIN and TMM must have 2n = 52 and that “Poque” and “Vitor” are individuals of F2 generation (2n = 50), it is possible that the hybrids described by De Oliveira et al. [9] (2n = 49) correspond to F3 as result of crossing between TMM and F2 hybrids with karyotype constitution similar to “Poque” (Figure 8A). Alternatively, it is also possible to propose that all the individuals analyzed in the present work and those studied by De Oliveira et al. [9] correspond to F1 generation of hybrids and that the differences in the number and type of chromosomes observed among them are the result of mechanisms of elimination (Figure 8B). Chromosome elimination is a process frequently described during the formation of hybrids in animals and plants, as a consequence of competition between parental genomes [39].

The chromosomal distribution of LINE-1 in TIN and TMM was similar to the pattern observed in *T. m. latirostris* [14]. In the latter species, LINE-1 was absent in the distal region of the short arm of the X chromosome, suggesting a possible role of this TE in the expression of pseudoautosomal genes [14]. This pattern was observed in TIN and TMM. The union of different genomes promoted by the hybridization process can lead to changes in the patterns of gene expression and DNA methylation [40,41]. In “Poque” FISH analysis revealed the greater number of LINE-1 signals, suggesting amplification of this element in the hybrid genome. The factors that promote high transcription of TEs in hybrids are poorly known and diverse; in F1 hybrid wallaby lines, for example, this phenomenon occurs through the hypomethylation of transposons [42], while in *Drosophila* (2) combination of different piRNA proteins in the hybrid genome, which may be functionally incompatible, or even have their biosynthesis is compromised due to the union of epistatic alleles of the genes that originate them [43]. Both mechanisms may explain our findings regarding “Poque”. The genomic instability caused by the activation and mobilization of TEs in hybrids can promote the emergence of new chromosomal rearrangements, as observed in marsupials [44]. In bats and rodents, LINE-1 is proposed to be involved in the genomic reorganization of these groups [45,46]. Considering this information, it is plausible to propose that the heteromorphism of pair 3 and the six chromosomes with unique banding patterns in the “Vitor’s” karyotype (which pairs could not be identified) may have their origin linked to new chromosomal rearrangements produced from of the activity of TEs during the hybridization process.

In our analyses, 45S rDNA sites were conserved, present in pair 20 in TIN, TMM, and “Poque”, in agreement with the Ag-NOR distribution described in previous cytogenetic studies [3,4,30]. According to Gray et al. [4], chromosome 20 of the Trichechidae family is conserved, as there is a similar pattern of G-bands for all the species mentioned above. In contrast, the distribution of U2 snDNA along the chromosomes demonstrates a high genomic dynamics of this sequence in *Trichechus*. Similar results have been reported for bony fish of the Batrachoididae [17] and Sciaenidae [47] families. Our findings for U2 snDNA dispersion can be explained by the association of this sequence with the transposable element (TE) Keno-1_SSa of the Tx-1 type, a specific element of U2 snDNA [48]. In *Characidium*, Pucci et al. [49], also evidenced the insertion of this transposable element in this multigene. Likewise, ET Utopia is also described as inserted into specific regions of U2 snDNAfrom different organisms [50]. Anjos et al., [51], when studying the genomic organization of U1 snDNAin grasshoppers, observed its association with SINE-like elements only in species that had multiple chromosomal loci of this multigene, suggesting its dispersion in the genome through transposition. Likewise, we propose that the action of transposable elements may have been an important factor in the dispersion of U2 snDNA in the *Trichechus* genome.

Chromosomal mapping of histone genes in animals usually shows the presence of one or more clusters of this multigene in the genome [52,53]. In TIN and TMM, histone H3 gene markings showed signals scattered along the chromosomes. The distribution of the pulverized signals may be associated with the insertion of the Helitron 4N1_SMo transposon in the Histone H3 sequence, as demonstrated by the sequencing analysis. In fact, this small segment similar to a Helitron element inner to the H3 probe could generate the localization of the Helitron-like copies on the karyotypes. On the other hand, this segment could be just a Helitron-similar stretch, without connection with the dispersion of the H3 copies visualized on Trichechus karyotypes. This is the first record of Helitron -Histone H3 association in mammals. Pucci et al. [49], suggested that TEs ERV1 and Gypsy, found in intergenic regions of histones H1 and H4, respectively, may be the cause of additional marking of these genes in the chromosomes of *Characidium*. The Helitron replication mechanism, known as rolling circle, comprises the capture and amplification of genomic fragments [54], generation of chimeric transcripts [55], and retention of DNA sequences that provide selective advantages [56]. The mobilization of genes or their constituent parts (exons, introns, regulators, etc.) through Helitron transposition has been reported in several eukaryotes [57,58]. In other organisms, such as chelonians and scorpions, it is suggested that histone H3 dispersion may be a consequence of the insertion of transposable elements [59,60]. Thus, it is plausible to suggest that the association Helitron -Histone H3 observed in *Trichechus* explains the pattern of the genomic organization of Histone H3 in TIN and TMM.

In conclusion, the variation observed between *T. inunguis* and *T. m. manatus*, mainly results from fusion-type rearrangements (based on current phylogenies of the Trichechidae family). Both species have a highly conserved 45S rDNA chromosome, while the multigenes U2 snDNA and histone H3 are dispersed in the chromosomes, showing a high dynamic distribution, which may be related to the insertion of TEs in these sequences. In the present study, we confirmed the hybrid identity of two *Trichechus* individuals, both with 2n = 50, however, with highly divergent karyotypes in relation to chromosomal structure. Our results will contribute to the establishment of strategies for breeding in captivity and releasing the animals and, consequently, for the conservation of Sirenians.

## Data Availability

Not applicable.
